# Nucleo-cytoplasmic transport of TDP-43 studied in real time: impaired microglia function leads to axonal spreading of TDP-43 in degenerating motor neurons

**DOI:** 10.1007/s00401-018-1875-2

**Published:** 2018-06-25

**Authors:** Adam J. Svahn, Emily K. Don, Andrew P. Badrock, Nicholas J. Cole, Manuel B. Graeber, Justin J. Yerbury, Roger Chung, Marco Morsch

**Affiliations:** 1 Department of Biomedical Sciences, Faculty of Medicine and Health Sciences, Centre for Motor Neuron Disease Research, Macquarie University, Sydney, NSW Australia; 2 Faculty of Life Sciences, The University of Manchester, Manchester, UK; 3 Brain Tumor Research Laboratories, Brain and Mind Centre, The University of Sydney, Sydney, NSW Australia; 4 Faculty of Science, Medicine and Health, School of Biological Sciences, University of Wollongong, Wollongong, NSW Australia

**Keywords:** Amyotrophic lateral sclerosis (ALS), Motor neuron disease (MND), Neurodegeneration, Microglia, TDP-43, Pathological spreading, Zebrafish

## Abstract

Transactivating DNA-binding protein-43 (TDP-43) deposits represent a typical finding in almost all ALS patients, more than half of FTLD patients and patients with several other neurodegenerative disorders. It appears that perturbation of nucleo-cytoplasmic transport is an important event in these conditions but the mechanistic role and the fate of TDP-43 during neuronal degeneration remain elusive. We have developed an experimental system for visualising the perturbed nucleocytoplasmic transport of neuronal TDP-43 at the single-cell level in vivo using zebrafish spinal cord. This approach enabled us to image TDP-43-expressing motor neurons before and after experimental initiation of cell death. We report the formation of mobile TDP-43 deposits within degenerating motor neurons, which are normally phagocytosed by microglia. However, when microglial cells were depleted, injury-induced motor neuron degeneration follows a characteristic process that includes TDP-43 redistribution into the cytoplasm, axon and extracellular space. This is the first demonstration of perturbed TDP-43 nucleocytoplasmic transport in vivo, and suggests that impairment in microglial phagocytosis of dying neurons may contribute towards the formation of pathological TDP-43 presentations in ALS and FTLD.

**Electronic supplementary material:**

The online version of this article (10.1007/s00401-018-1875-2) contains supplementary material, which is available to authorized users.

## Introduction

Pathological deposition of protein aggregates characterises a large group of neurodegenerative diseases in which the key event is considered to be either the malformation of a key protein, or the failing of a crucial metabolic cascade, leading to the generation of a pathological proteinaceous agent that can self-propagate and result in gain- or loss-of-function progressing toward cell death [38].

An aggregating proteinopathy involving the TAR DNA-binding protein (TDP-43) provides a molecular link between two progressive neurodegenerative diseases: amyotrophic lateral sclerosis (ALS) of the motor cortex, brainstem and spinal cord, and frontotemporal lobar degeneration (FTLD) of the frontal, insular, and temporal cortex (non-SOD1 ALS and non-tau FTLD, termed ALS-FTLD; [3, 13, 15, 22, 43]).

In biochemical and cell culture models, TDP-43 has been identified as spontaneously aggregation prone [1, 27]. This propensity has been localised to a low-complexity domain in the C-terminal region that may play a role in RNA binding, granule formation, and/or liquid–liquid phase separation to subserve transcription dynamics and mRNA transport [6, 12, 32, 34, 55, 56]. It is hypothesised that inherited or spontaneous mutation of the encoding gene, *TARDBP*, or of an interacting partner, upsets the balance between aggregation and dissolution of TDP-43 [14, 71], which may be differentially regulated under changing conditions, including during the cellular stress/death response [7, 30].

TDP-43 also appears to display self-propagating behaviour. Feiler et al. [21] found that naive cultured mouse neurons will internalise tagged, oligomerized TDP-43 from the culture medium of transfected neurons. This was mediated by the packaging of TDP-43 into microvesicles or exosomes, which were more effective at transmission than free protein. Linking this phenomenon to clinical observations, when cell culture models were seeded with phosphorylated TDP-43^+ve^ insoluble fractions from ALS and FTLD patient brain or spinal cord, there was a significant increase in intracellular endogenous TDP-43 aggregation and subsequent cell death [44, 57]. To follow on from this we examined how motor neuron injury and subsequent degeneration mobilises TDP-43 in vivo and may contribute to pathogenicity in ALS.

We have developed an in vivo transgenic model to visualise subcellular compartments of individual motor neurons in zebrafish spinal cord. Using this new model, we have expressed fluorophore-tagged human TDP-43 within individual spinal motor neurons to observe the intraneuronal localisation of TDP-43 over time. After confirming that the subcellular localisation of TDP-43 is the same in zebrafish and human, we discovered in vivo that TDP-43 concentrated in highly dynamic nuclear granules with droplet-like properties. By application of a cell-specific UV injury, we were able to induce neuronal degeneration, within which the fate of TDP-43 could be precisely followed. We found that motor neurons underwent a stereotyped neurodegenerative process with structural changes closely mirroring those of degenerating neurons in human tissue. We further report upon live-imaging evidence for different pathways of TDP-43 release and distribution; as aggregated fragments, diffuse in cytoplasm, as a component of pyknotic or karryhorexic nuclear fragmentation, anterograde axonal diffusion or as a component of microglial uptake.

## Materials and methods

### Zebrafish care

Zebrafish (*Danio rerio*) were maintained under standard conditions [65]. Experiments were conducted under Macquarie University Animal Ethics and Biosafety approvals (2012/050 and 2015/033; 5201401007). Larvae were raised in E3 medium at 28 °C on a 14:10 light:dark cycle. 1-Phenyl-2-thiourea (PTU) was added to the E3 medium at 24 hpf at 0.005% to inhibit pigmentation. All experiments in this study were conducted at 3–5 days post-fertilisation (dpf).

### Transgenes

*Tg(*-*3mnx1:kalTA4*-*4xnrUAS*-*E1b*-*H2B*-*mCerulean3*-*P2A*-*mKO2*-*CAAX)* (MQ13) was generated using recombined p5E-3mnx1 [40], pME: kalTA4-4xnrUAS-E1b, p3E-H2B-mCerulean3-P2A-mKOFP2-CAAX [17], and pTol2pA2 [33]. The pME:kalTA4-4xnrUAS-E1b construct was generated from PCR amplification of *Eco*RI- and *Xho*1-flanked kalTA4, and *Xho*1- and *Sal*1-flanked 4xnrUAS sequences from pME:kalTA4 and pME:4xnrUAS vectors, respectively (both vectors were a kind gift from the Parton Lab, University of Queensland). A *Sal*1- and *Spe*1-flanked E1b minimal promoter was added to the 3ʹ end and the construct subcloned into a lab derived pDONR 221 (Invitrogen) vector with *Eco*RI and *Spe*1 sites. The codon-optimised H2B–mCerulean3–P2A–mKOFP2–CAAX sequence was ordered from GeneArt and recombined into pDONRP2R-P3 (Invitrogen) to create the p3E–H2B–mCerulean3–P2A–mKOFP2–CAAX [17]. It incorporated a nuclear-localised mCerulean3 fluorophore and a membrane-localised mKOFP2 (mKO2) fluorophore expressed in motor neurons.

*Tg (*-*3mnx1:eGFP*-*HsaTDP*-*43*^*WT*^*)* was generated using recombined p5E-3mnx1 (*#74632*) [40], pME-EGFP [33], p3E-HsaTDP-43 and pTol2pA2 [33]. The p3E-HsaTDP-43 was generated by subcloning a *Bam*HI–*Spe*1-flanked gene string encoding HsaTDP-43 (GeneArt) into the *Bam*HI–*Spe*1 sites of p3E-MCS (*#75174*) [17].

Transgenic lines were generated by co-injection of the Tol2-flanked constructs and transposase [28].

### PU.1 (spi1b) morpholino

The PU.1 morpholino and a mismatch control used in this study were the same as those of Rhodes et al. [50], obtained from Gene Tools. Injections consisted of a 2 ng/nl concentration of either active or mismatch morpholino in a 2-nl droplet calibrated by micrometre. Immediately after injections, dishes containing equal numbers of either active or mismatch injected larvae or uninjected siblings were coded by a third party and all following analyses were conducted blind to the injection condition. At 3 and 5 dpf, larvae were screened for developmental abnormalities and for the presence of any mCherry-CAAX fluorophore expression, indicating the presence of mpeg1 expressing monocytes.

- PU.1 (spi1b)-targeted active MO: 5ʹ-GATATACTGATACTCCATTGGTGGT-3ʹ
- PU.1 (spi1b) mismatch MO: 5ʹ-GATAAACTGTTACTCGATTGCTGGT-3ʹ

### Notes on morpholino and zebrafish PU.1 orthologues spi1a/b

An in silico analysis showed that for the two zebrafish orthologues of PU.1, spi1a and spi1b, the morpholino above contains a matching sequence only for spi1b (*D. rerio*, GRCz10). Given the efficacy of the morpholino in this and previous studies [25, 48–50] knockdown of spi1b appears sufficient to induce primitive monocyte lineage inhibition in the zebrafish.

### Imaging and UV injury induction

Imaging was conducted on a Leica SP5 confocal microscope. mTagBFP was excited by 405-nm laser diode, which was also used at higher power to induce UV injury [41]. eGFP was excited by an argon laser at 488 nm. mKO2-CAAX and mCherry-CAAX were excited by a Leica white-light laser at 552 and 587 nm, respectively. Imaging for Fig. 2 and Online Resources 1 and 6 was conducted with an 8 kHz Leica resonance scanner. Objectives used were a Leica 40x HCX APO L U-V-I water immersion and a Leica 63X HC APO U-V-I CS2 water immersion.

**Fig. 1:**
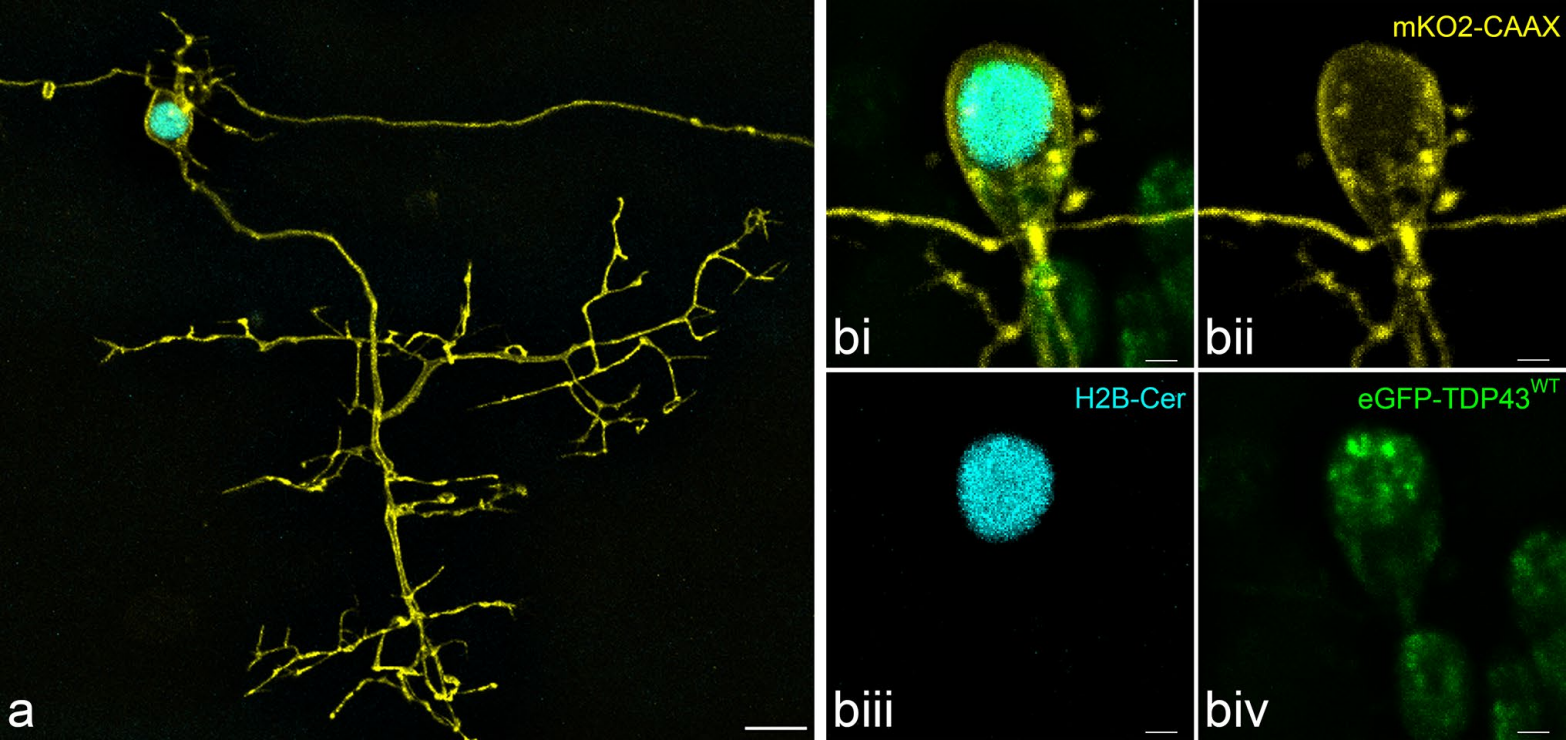
Fluorescent primary motor neurons in the zebrafish spinal cord at 3 dpf. **a** Maximum intensity projection of a z-stack of a motor neuron revealing the complete axonal arbour (yellow) projecting into and around the myotome (not shown). At the cell body the spindly dendritic arbour can also be observed. The nucleus is visualised in mCerulean3 (cyan). Traversing the image is the axon of a spinal interneuron. Scale = 10 µm. **b** A separate fluorescent motor neuron (**bi**), demonstrating the mnx1-driven membrane-bound mKO2-CAAX (yellow, **bii**), nuclear H2B-mCerulean3 (cyan, **biii**) and HsaTDP43^WT^-linked eGFP (green, biv). Scale = 2 µm

**Fig. 2:**
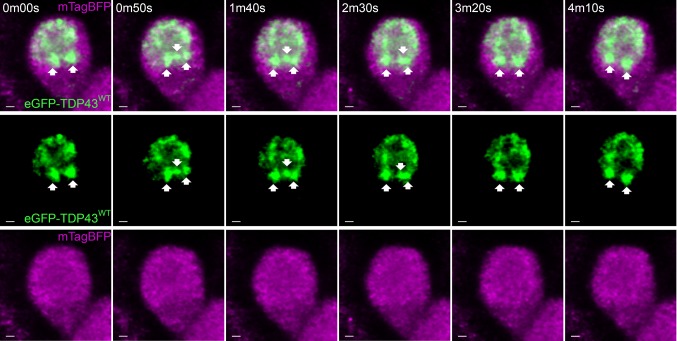
Nuclear TDP-43 accumulations are highly motile. Fast resonance scan of a motor neuron cell body with cytoplasmic mTagBFP (magenta) and nuclear eGFP-TDP43^WT^ (green) revealed that these TDP-43 accumulations rapidly dissociate and coalesce. The full time-lapse video gives the best example of the TDP-43 motility (Online Resource 1). Arrows indicate and follow distinct accumulations over the imaging period. Scale = 1 µm

Imaging processing was conducted in ImageJ (1.51n). Deconvolution was applied for Figs. 1, 2, 4, 5, 6 and 7. Point spread functions were generated by the Richards and Wolf algorithm [51] as implemented in the PSF generator plugin for ImageJ [31]. Richardson–Lucy deconvolution was carried out in the DeconvolutionLab plugin for ImageJ [53]. A mean filter of radius 1 was applied to Figs. 4 and 6.

### 3D volume analysis of TDP-43 nuclear/cytoplasm ratio

The ratio of nuclear to cytoplasmic eGFP-TDP43^WT^ over the 3D neuron volume was derived by first establishing a separation of voxels containing eGFP-TDP43^WT^ alone (cytoplasm) or both eGFP-TDP43^WT^ and H2B-mCerulean3 (nucleus) using a custom macro in ImageJ. Fluorescence grey value of each voxel was regarded as an indication of fluorophore concentration and the voxel grey value was summed over each set. The neurons used in this analysis were imaged from the same crossing and with identical microscope excitation and collection settings.

### 3D volume analysis of TDP-43 accumulations

The volume measurements of the nuclear accumulations of eGFP-TDP43^WT^ were derived by automatic 3D object recognition in ImageJ. 205-µm × 51-µm × 24-µm z-stacks were acquired in the spinal cord of three larvae expressing mnx1-driven eGFP-TDP43^WT^ from the same crossing. Object counting was conducted with the Object Counter3D plugin in ImageJ (plugin authors are F. Cordelières and J. Jackson. The plugin is available at http://rsbweb.nih.gov/ij/plugins/track/objects.html).

### Axonal eGFP localisation

For the analysis of eGFP in the axon before and after UV injury of Fig. 7 and Online Resource 9, the Simple Neurite Tracer plugin for ImageJ (v3.1.3, [36]) was used to generate a neurite vector and gather individual segment and whole neurite fluorescence intensity measures.

For the baseline of eGFP signal present in uninjured motor neuron axons of Supplementary Figure 4 (Online Resource 10), an 8-µm segmented line was drawn along the axon path from the hillock in Z-projected stacks, as visualised by the mKO2-CAAX fluorophore. The mean grey value was measured in the voxels falling on this line for both mKO2-CAAX and eGFP. To demonstrate that any eGFP signal was not lost to a process of background subtraction, we sampled two 8-µm lines in regions of the same image without a positively expressing cell to determine the background levels for each fluorophore in each image. These background values were averaged and reported to give the background value used for comparison to the axon path. The neurons used in this analysis were imaged from the same crossing and with identical microscope excitation and collection settings.

### Statistical analysis

The software R (3.3.2) was used for statistical analyses. Differences between the means were evaluated using ANOVAs and post hoc Tukey tests. For all statistical tests, significance was taken as *P* < 0.05. Unless otherwise indicated, data values are presented as the mean ± standard error of the mean (SEM). Graphs were generated in R (3.3.2) and Microsoft Excel 2016.

## Results

### Visualising the distribution of TDP-43 within spinal motor neurons using sub-compartment-specific reporters

To visualise and temporally monitor the process of motor neuron degeneration and the subsequent fate of intraneuronal TDP-43 in the living spinal cord, we developed a multi-transgenic approach in zebrafish. Expression of fluorophore reporters was driven by the *mnx1* promoter, previously described in primary motor neurons and interneurons in the zebrafish spinal cord [40, 42, 68]. Fluorophores were targeted towards specific subcellular compartments through the use of specific peptide tags, for membrane (mKO2-CAAX), nucleus (H2B-mCerulean3) and cytoplasm (mTagBFP) (Figs. 1, 2). Human wildtype TDP-43 (TDP-43^WT^) was visualised with an eGFP tag (eGFP-TDP43^WT^, Fig. 1biv).

In the motor neurons of the zebrafish spinal cord, eGFP-TDP43^WT^ was observed to be strongly concentrated in the nucleus while relatively diffuse in the cytosol (Figs. 1biv, 2). This is in keeping with qualitative observations in human tissue section [43, 61], mouse tissue [39, 54] and cell culture models [4, 64]. To quantify this distribution, we assayed the relative distribution of eGFP-TDP43^WT^ across the nucleus and cytoplasm over the entire cell body as a 3D volume, as determined by comparison with the co-expressed nuclear marker, H2B-mCerulean3 (see “Materials and methods”). We found that 78.2% of eGFP-TDP43^WT^ localised to the nucleus (± 4.6%, *n* = 12 neurons in 4 larvae). This percentage is in line with a ~ 85% nuclear localisation reported previously using western blot quantitation of nuclear/cytoplasmic fractions from cultured hippocampal neurons [64].

### Visualisation of dynamic TDP-43 granula in the nucleus of spinal motor neurons

The nucleus contained eGFP-TDP43^WT^ in the form of discrete fluorescent “granula”. Automatic 3D object analysis of the TDP-43 aggregates revealed a volume range of 0.023–5.7 µm^3^, with 83% falling in the range of 0.023–0.048 µm^3^ (132 accumulations in three larvae, Supplementary Fig. 1 (Online Resource 10)). Live imaging revealed that these TDP-43 granula were not stationary but showed highly dynamic movements within the nucleus (Online Resource 1). Using high-frequency image acquisition (8 kHz resonance scanner, image every 50 s), droplet-like interactions between the accumulations could be observed (Fig. 2 and Online Resource 1). These may be indicative of TDP-43 incorporation in nuclear phase-separation compartmentalisation [60, 70].

### Targeted UV-mediated injury in motor neurons induced a local microglial response including phagocytosis of intracellular TDP-43

We have previously demonstrated that application of a UV laser (405 nm) can selectively induce injury and cell death in zebrafish motor neurons in vivo [40, 41]. The UV laser beam can be focused to a narrow column of subcellular width. Accordingly, as shown in experiments with spinal cord motor neurons expressing the photoconvertible Kaede fluorophore, a single motor neuron can be precisely targeted [40]. To further characterise the UV-mediated cellular damage in this in vivo model, we examined the bleaching curves in the regions immediately neighbouring the laser beam. Bleaching did occur around the target column; however, it was attenuated by 50% within 10 µm (approx. one cell width) and nearly 80% within 20 µm [Supplementary Fig. 2 (Online Resource 10)]. This steep power attenuation in the immediate surroundings of the laser beam supports the view that our method can be used to inflict very precise subcellular damage to selectively induce degeneration of a TDP-43-expressing neuron without inducing any significant injury to surrounding cells.

To examine the response of spinal microglia to the degeneration of a single spinal motor neuron, we generated transgenic fish that co-express cytoplasmic mTagBFP in motor neurons [17] and mCherry-CAAX in macrophages/microglia (mpeg1.1 promoter, [18]), respectively. Using DNA injections of eGFP-TDP43^WT^ into these larvae we achieved expression of tagged TDP-43 in individual spinal motor neurons that allowed precise visualisation of TDP-43 distribution after laser ablation (*n* = 7 motor neurons in 6 larvae).

**Fig. 3:**
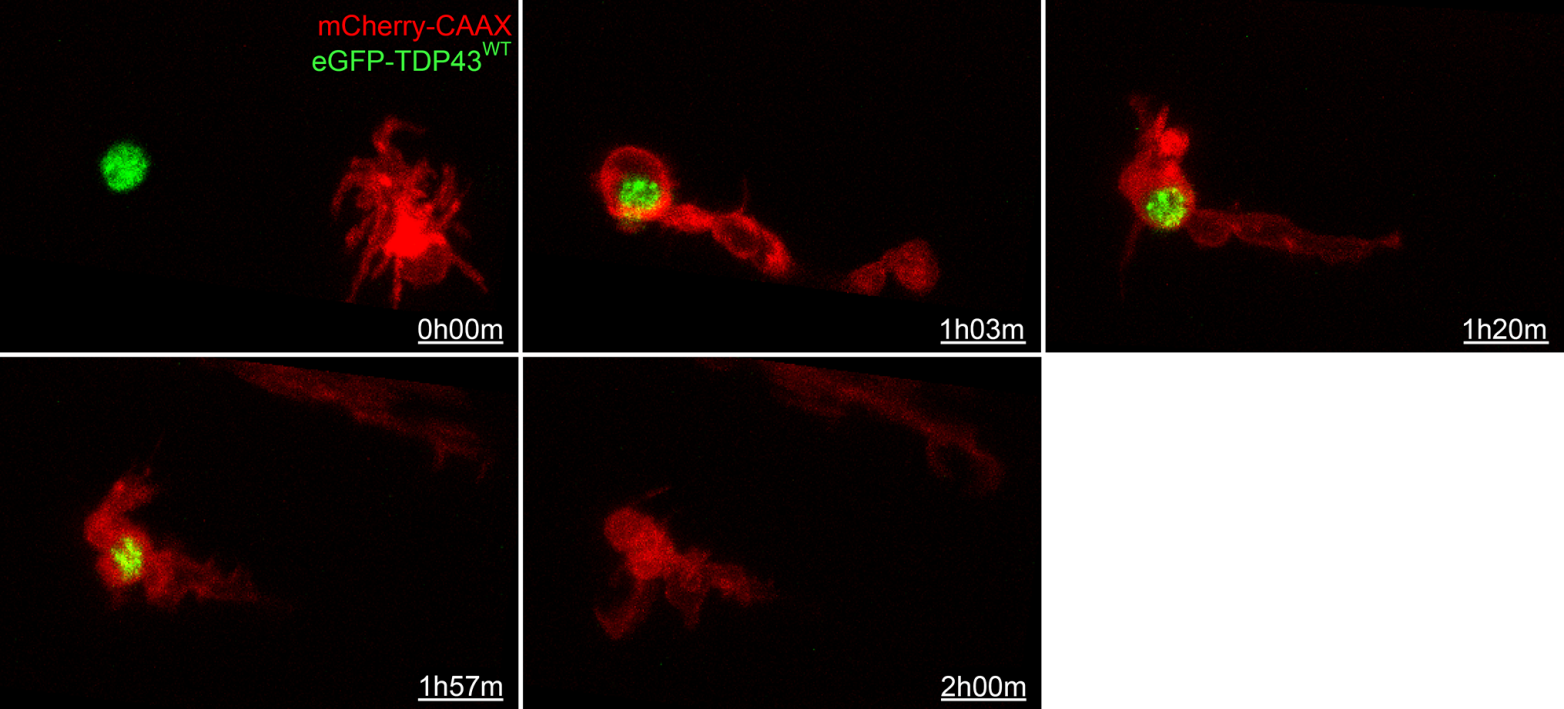
Microglial uptake of TDP-43. Sequence of a spinal microglia (mCherry-CAAX, red) migrating toward and engulfing an eGFP-TDP43^WT^ (green)-expressing motor neuron during UV-induced degeneration. Scale = 10 µm

Following targeted injury, individual spinal microglia could be observed to approach the injured neuron either to phagocytose the cell soma (*n* = 3; Fig. 3 and Online Resource 2) or to establish contact without phagocytosis (*n* = 3; Online Resource 3; one other neuron showed no morphology change or microglia response within a 6 h imaging period). The microglia response was swift and occurred within 1–2 h post-injury. The response was directed as the microglia cell body moved towards the injury site and extended cell processes towards the target membrane. We have previously characterised and quantified this phagocytic behaviour in more detail [40]. Overall we confirmed that UV-induced neuron degeneration triggers a local microglia response while sub-lethal stress results in a transient microglia interaction (Online Resource 3).

In three experiments in which we observed microglia phagocytosis of targeted neurons, the microglia ingested TDP-43 as part of their phagocytic response. Visualising the localisation of eGFP-TDP43^WT^ during this process showed that during laser-induced neuronal degeneration microglia internalised TDP-43 predominantly as a component of the neuron nucleus. The fluorescence was lost rapidly (between frames, < 4 min) in the microglial phagosome, likely due to lysosomal fusion and rapid acidification of the organelle [48]. This is reminiscent of the finding in the zebrafish tectum that microglia phagocytosis occurs early in the cell death process, usually preceding the binding of extracellular AnnexinV [48, 62] and suggests that microglia play an important role in phagocytosing degenerating motor neurons and intraneuronal TDP-43.

### Depletion of microglia revealed the sequence of neurodegeneration and TDP-43 redistribution

To fully elucidate the fate of TDP-43 in a dying motor neuron in vivo, we inhibited microglial phagocytosis by morpholino targeting of PU.1 (zebrafish spi1b, see “Materials and methods”). PU.1 is a well-characterised transcription factor and essential for monocyte to macrophage lineage progression, and knockdown of PU.1 has been shown to reliably cause macrophage deficiency in zebrafish [25, 48–50].

Larvae were injected with a PU.1-targeted morpholino or a 4-bp-mismatch control and screened for effective macrophage knockdown as well as common morpholino-associated developmental abnormalities [5]. Specific morpholino-induced abnormalities were not observed [Supplementary Fig. 3a (Online Resource 10)] and macrophage knockdown was confirmed at 72 h post-fertilisation (hpf) [Supplementary Fig. 3b (Online Resource 10)]. Repopulation of macrophages in these knockdown larvae had begun at 120 hpf.

**Fig. 4:**
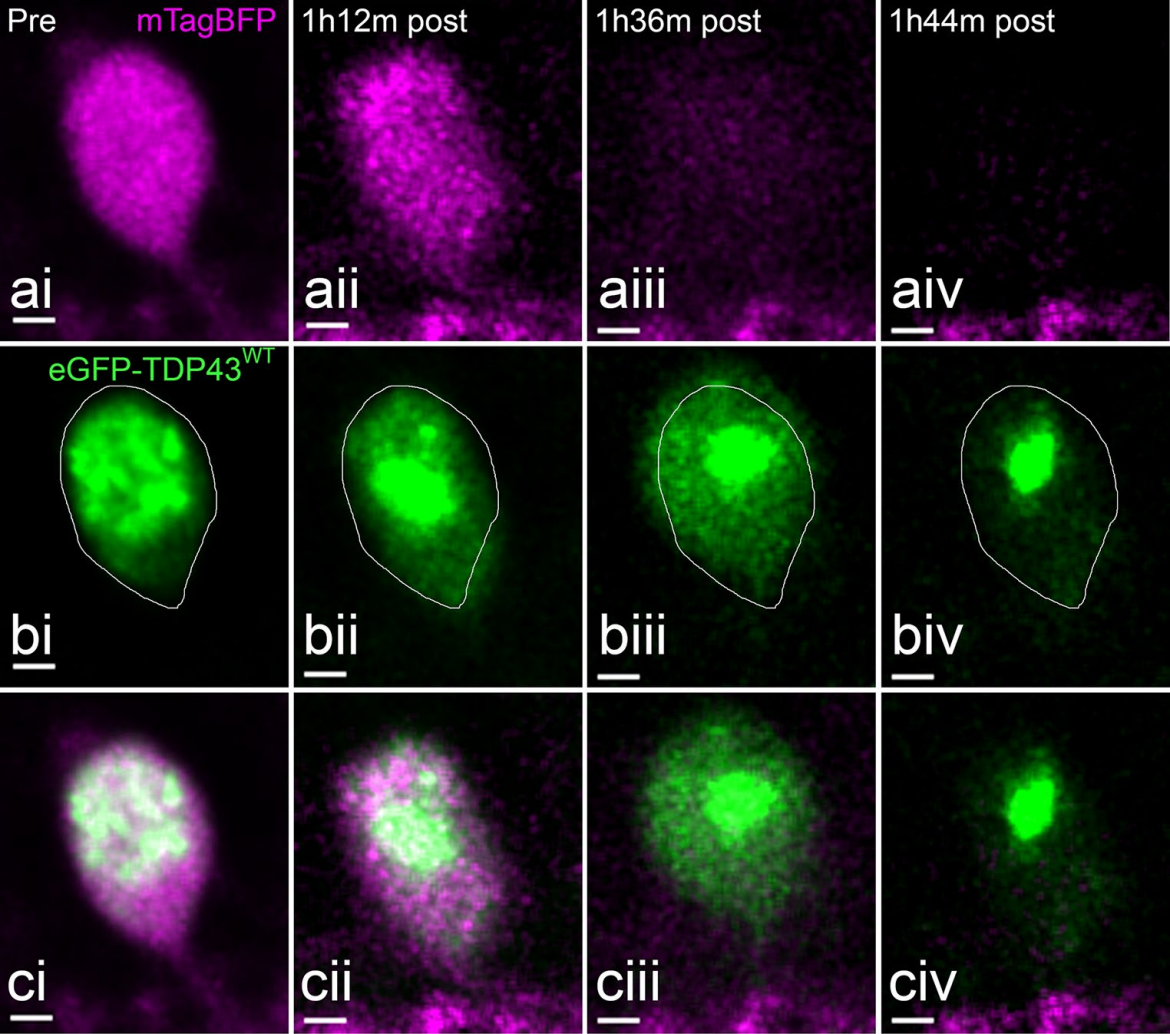
Expansion and dissolution of a motor neuron over the course of UV-induced degeneration in the absence of microglia. **ai** Cytoplasmic mTagBFP signal pre-irradiation. **aii**–**iv** Cytoplasmic changes during UV-induced degeneration. **bi** eGFP-TDP43^WT^ distribution in the soma of the neuron pre-irradiation. **bii**–**iv** eGFP-TDP43^WT^ mislocalises into the cytoplasm during degeneration. **c** Merged images. Scale = 2 µm

**Fig. 5:**
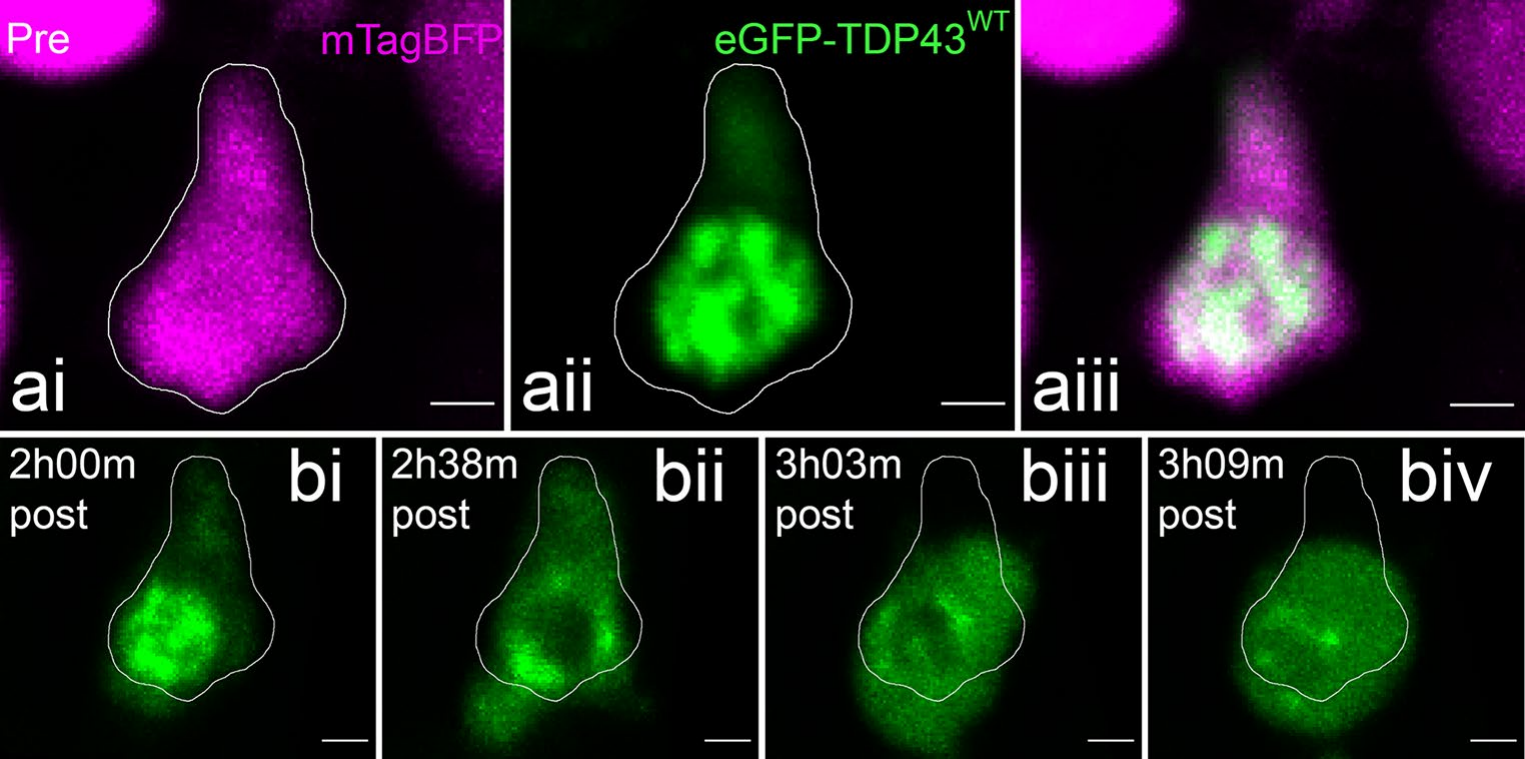
Blebbing and TDP-43 mislocalisation in a motor neuron over the course of UV-induced degeneration after depletion of microglia. **ai** Cytoplasmic mTagBFP signal pre-irradiation. **aii** eGFP-TDP43^WT^ distribution in the soma of the neuron pre-irradiation. **aiii** Merged images. **bi**–**iv** eGFP-TDP43^WT^ signal redistributes into the cytoplasm and shows the blebbing of the cell soma during degeneration. Note the apparent fragmentation of the nucleus, in contrast to the concentration of nuclear eGFP-TDP43^WT^ in the previous figure. Scale = 2 µm

Within the 72–120-hpf time window, spinal cord motor neurons were injured with the laser as described (*n* = 14 cells in 12 larvae). In the absence of the primary macrophages, targeted motor neurons underwent a consistent process of degeneration. Following injury, the motor neuron cell bodies showed swelling (*n* = 10 of 14, Figs. 4, 5). At its maximum, the cell became spherical in shape (Figs. 4ciii, 5biv, 6ci, 7biv). The time taken to reach this full expansion was variable (22 min to 5 h 26 min). However, within minutes of reaching this morphology, all neurons rapidly shrunk and lost the majority of the cytoplasmic fluorescent signal as it became dispersed to the extracellular space (Online Resources 4 and 5).

**Fig. 6:**
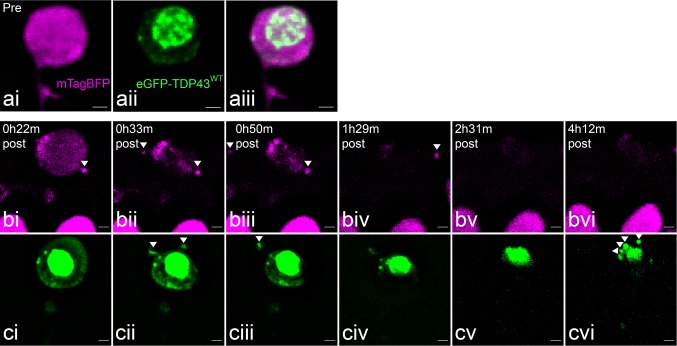
Fragment release from a neuron undergoing UV-induced degeneration in the absence of functional microglia. **ai** Cytoplasmic mTagBFP signal pre-irradiation. **aii** eGFP-TDP43^WT^ distribution in the soma pre-irradiation. **aiii** Merged images. **bi**–**vi** mTagBFP during UV-induced degeneration. Concentrated fragments can be observed separating from the external membrane before the cytoplasm disperses. **ci**–**vi** eGFP-TDP43^WT^ during degeneration. Concentrated fragments can be observed collecting and releasing from the membrane. The concentrated (pyknotic) nuclear core gradually fragments. Scale = 2 µm

**Fig. 7:**
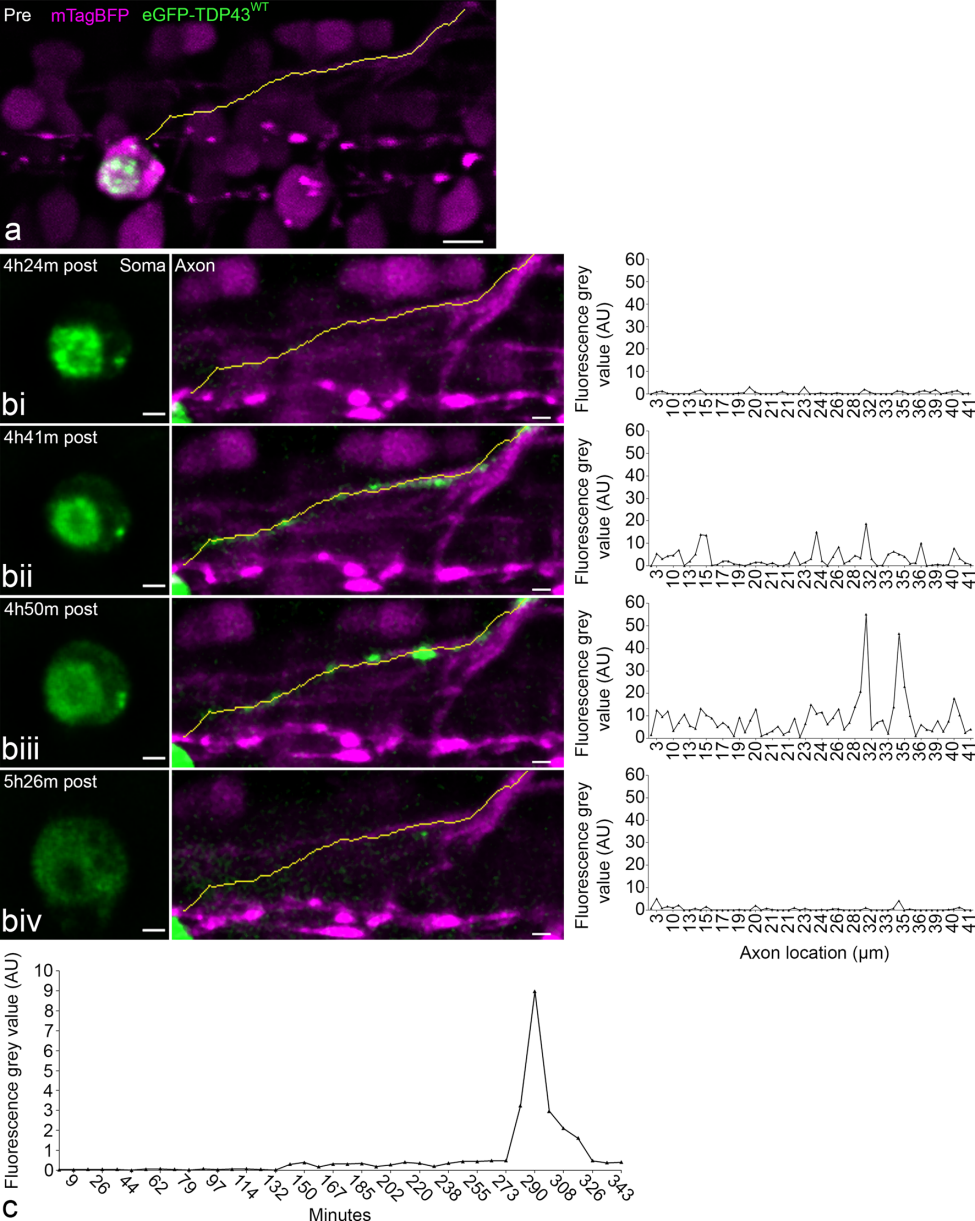
Axonal redistribution of eGFP-TDP43^WT^ during UV-induced degeneration and microglia depletion. **a** Pre-irradiation, line is a projection of the axon vector used for signal analysis. Scale = 5 µm. **bi**–**iv** At each time point from left to right the three windows represents a single time point illustrating the cell body and the TDP-43 distribution (soma, eGFP-TDP43^WT^ in green), the axonal projection (axon, mTagBFP in magenta incl. neurite vector as yellow line), and the fluorescence intensity of eGFP in the segments along the neurite vector. **c** Line graph demonstrating the time course of the mean eGFP fluorescence intensity along the axon post-stress induction. Scale = 2 µm. Note: axonal redistribution was observed in 2 of the 10 neurons, which were targeted for UV injury and underwent degeneration

Importantly, during the phase of cellular expansion, eGFP-TDP43^WT^ rapidly redistributed from the nucleus to the cytoplasm (Figs. 4b, 5b). This process was confirmed using high-frequency image acquisition (8 kHz resonance scanner sequence, Online Resource 6). The nucleus remained visible as the brightest accumulation of eGFP-TDP43^WT^ in either a condensed form (pyknotic) (Fig. 4biii) or it became fragmented (karyorrhexic) (Fig. 5 bii–iv). In the case of pyknosis, the remaining core of nuclear eGFP-TDP43^WT^ persisted initially and disintegrated slowly thereafter (see next section).

### TDP-43 fragments are released by dying motor neurons

Prior to the expansion and dissolution of the degenerating neuron, and in the absence of phagocytosing microglia, we occasionally observed fluorescently tagged fragments moving from the cytoplasm into the extracellular space. As shown in Fig. 6 and Online Resource 7, these fragments occasionally contained eGFP-TDP43^WT^. After expansion and dissolution, and after the nucleus had assumed a pyknotic morphology, the remaining core of eGFP-TDP43^WT^ could be observed to gradually fragment and disperse (Fig. 6cvi and best illustrated live in Online Resource 7).

### Axonal mislocalisation of TDP-43 prior to cellular disintegration

In addition to morphological alterations of the cell body, we also observed beading and disintegration of the axon as a sequela of UV-induced degeneration of spinal motor neurons (Online Resource 8). To follow up on this, we investigated whether the mislocalisation of TDP-43 into the cytoplasm was accompanied by an increase in axonal TDP-43. To establish a baseline, we examined eGFP-TDP43^WT^ distribution in the axons of uninjured neurons in mKO2-CAAX co-expressing larvae. eGFP-TDP43^WT^ was not detected along the axon proximal to the cell body [*n* = 8 neurons in three larvae, Supplementary Fig. 4 (Online Resource 10)]. Similarly, for the motor neurons targeted by UV to induce degeneration, such as the neuron demonstrated in Fig. 7, no eGFP-TDP43^WT^ was detectable along the motor axon from the hillock to its conjunction with the axon bundle leading down into the myotome, whereas mTagBFP was present throughout the cytoplasm and along the axon. However, in two of the ten motor neurons that underwent UV-induced degeneration, we observed that the process of cellular expansion was accompanied by a rapid movement of eGFP-TDP43^WT^ into the axon. The process is illustrated in Fig. 7 and Online Resource 9, both visually and by plotting the eGFP fluorescence intensity along the 3D traced axon over time. Following injury, the mean eGFP-TDP43^WT^ intensity along the axon increased during the process of degeneration before spiking rapidly ~ 5 h after the initial UV stress (but prior to dissolution as described above). The observation of inducible spreading of TDP-43 into axons in a living organism points to the existence of a potential molecular mechanism for progressive transmission and redistribution of pathological TDP-43 to neighbouring and distal neurons within the motor system.

## Discussion

**Fig. 8:**
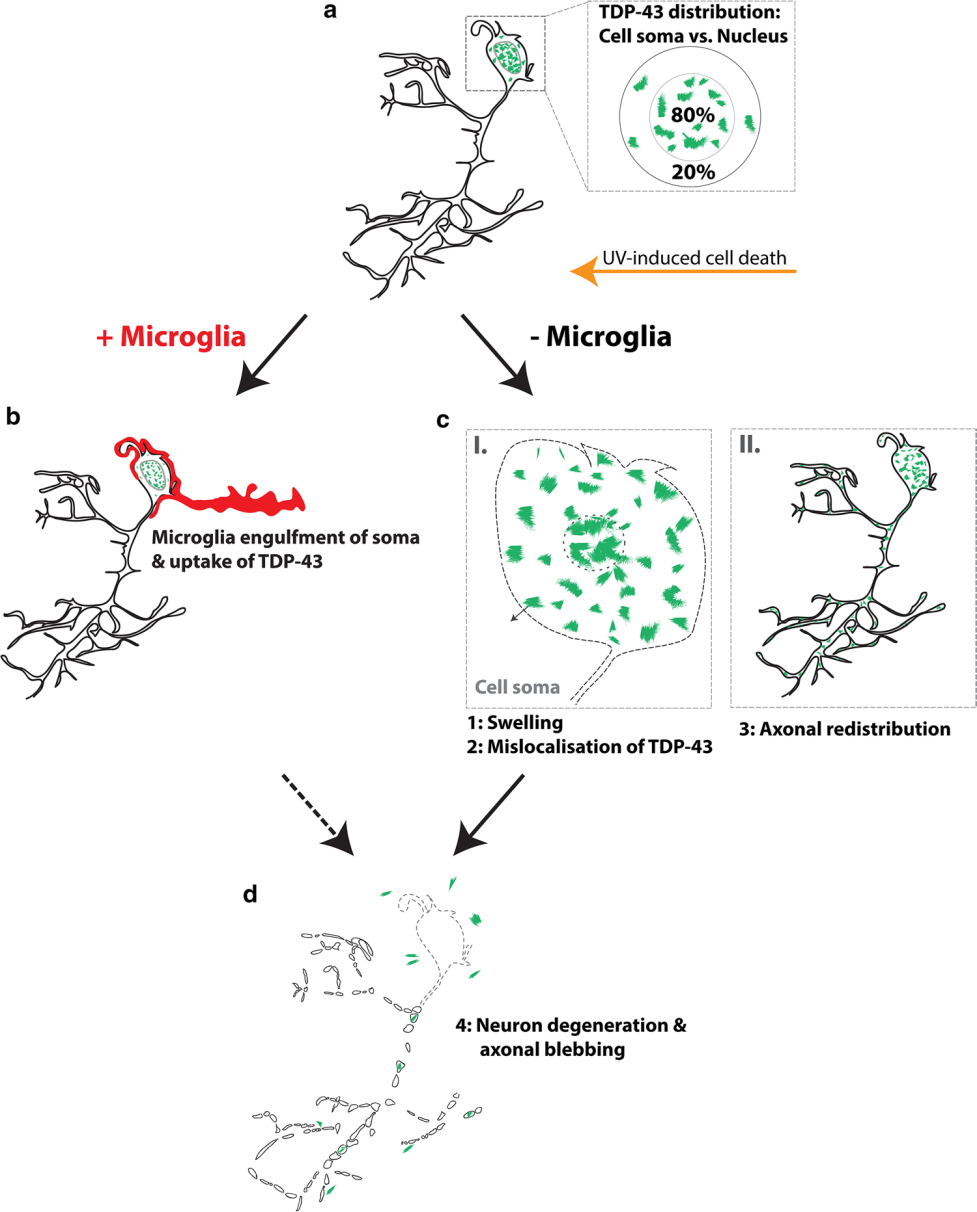
Illustration of the proposed in vivo process of stress-induced degeneration of a neuron in the zebrafish spinal cord. **a** An uninjured spinal motor neuron expressing fluorescent TDP-43 (green) predominantly in the nucleus. Insert depicts the measured distribution of TDP-43 between the cytoplasmic and nuclear compartments in the cell soma volume with ~ 80% TDP-43 localised in the nucleus (dotted line) and ~ 20% localised in the cytoplasm. **b** In the presence of microglia (red), the stressed/injured neuron is rapidly recognised and phagocytosed by the microglial cell. TDP-43 is taken up and contained during this process. **c** In the absence of microglia, the stressed/injured neuron undergoes a characteristic sequence of neurodegeneration and TDP-43 redistribution (I–II). Individual stages of this process have been described previously in clinical literature and are cited below (note that the continuous sequence of events has so far not been demonstrated in vivo). The cell soma shows dysmorphia with a distinctive swelling (dotted line, resembling expansion or “ballooning” observed across neurodegenerative histology: AD and CJD [16], SMA [37], HD [52], TDP-43 mislocalisation into the cytoplasm [23] and nuclear condensation [24]). At the end stage of this process TDP-43 is redistributed along the axonal projections into the distal parts of the neuron [9–11]. **d** The degenerated neuron loses its integrity initially in the cell soma and shows characteristic blebbing of the degenerating axonal projections. Conceivably, the lack of neuroprotective microglia allows the spread of TDP-43 aggregates into the neuropil and extracellular tissue

Our understanding of the molecular pathogenesis of ALS and FTLD is limited by our incomplete understanding of the sequence of events underpinning the pathology. Many of the insights we currently have on the hallmarks of ALS/FTLD were derived from postmortem histological studies. In almost all ALS patients and more than half of FTLD patients, the key molecular histopathological hallmark is the presence of abnormal amounts of intracellularly deposited TDP-43. It typically forms cytoplasmic-insoluble aggregates within neurons [3, 43]. Numerous in vitro models of TDP-43 overexpression (wildtype and mutant) have been investigated to understand how these TDP-43 aggregates are formed. These studies collectively suggest that TDP-43 is a highly aggregation-prone protein [27] and that a variety of molecular pathways can lead to cytoplasmic mislocalisation, aggregation and inclusion formation [14, 20, 30, 56, 71]. Moreover, although a true phenocopy of ALS/FTLD has been elusive in TDP-43 animal models [26], the overexpression of mutant forms of TDP-43 in mice does result in the development of motor impairment and lethality [29, 58, 63]. A key challenge with these animal models is that similar to the clinical postmortem situation, the disease histopathology only provides snapshots at different time points during disease progression. In contrast, in the present study, we were able to observe the fate and dynamics of TDP-43 cytoplasmic deposition in real-time at high resolution within the spinal cord in vivo. Notably, we were able to identify distinct stages of motor neuron degeneration and the mislocalisation and spread of TDP-43. In Fig. 8, we summarise the temporal sequence of neurodegeneration and TDP-43 mislocalisation. While this process of UV-mediated injury can reflect an accelerated cell-death pathway, we occasionally observed the formation of TDP-43 aggregates in the cytoplasm of spinal motor neurons. It will be important to explore if these aggregates are of similar structure and function to the pathological aggregates (e.g. phosphorylation status of TDP-43) that are observed in diseased human postmortem tissue and in animal models overexpressing mutant TDP-43.

The subcellular redistribution of TDP-43 from the nucleus to the cytoplasm in injured motor neurons (when microglia were depleted and, therefore, the cells were not rapidly phagocytosed) was especially striking, and this study represents the first real-time in vivo observation of the phenomenon. This redistribution of TDP-43 to the cytoplasm has been suggested to represent a landmark feature of the onset and progression of ALS/FTLD [23, 43], and in cell culture the disrupted trafficking of TDP-43 is a precursor to the formation of cytoplasmic TDP-43 inclusions [66]. Previously, Sato et al., [54] have reported the cytoplasmic mislocalisation of TDP-43 in response to neuronal stress in a mouse ligation model. Our observations provide in vivo support for the hypothesis that cytoplasmic accumulation of TDP-43 in motor neurons could occur as a direct downstream consequence of neuronal stress or injury. It is important to point out that in our experiments microglia did not directly prevent the cytoplasmic mislocalisation of TDP-43 in UV-injured motor neurons, rather this process was circumvented by the microglial phagocytosis of the degenerating neuron. This raises an intriguing question—why is TDP-43 pathology limited to ALS/FTLD and not other neurodegenerative diseases (such as Alzheimer’s, Parkinson’s and Huntington diseases)? It is possible that the TDP-43 redistribution that we have observed is elicited by specific types of neuronal stress/injury (such as UV that was used in this study). Alternatively, it is possible that microglia are specifically defective in their phagocytic function in ALS/FTLD (and not other diseases), therefore, allowing intracellular TDP-43 redistribution to occur. These are important directions for future follow-up. Interestingly, Spiller and colleagues recently demonstrated the selective uptake of TDP-43 by reactive microglia in mice, and that this process is likely to play an important role in clearing pathological (mislocalised) TDP-43 from neurons [59].

Significant recent interest in the field has focussed on the potential cell-to-cell transfer of TDP-43 and/or associated proteins, and the possibility that this might facilitate propagation of the disease process through the motor system. In vitro, the intercellular transfer of TDP-43 has been demonstrated [21, 47, 57, 67]. However, whether this also occurs in vivo remains an open question. In our study, we observed two pathways for transfer of TDP-43 from the originating dying neuron: uptake by microglia via direct phagocytosis and transfer into the surrounding neuropil.

UV injury-induced degeneration resulted in a phagocytic response by spinal microglia, which took up predominantly nuclear TDP-43. This represents the first in vivo demonstration of TDP-43 uptake by microglia as well as the prevention of TDP-43 spreading by normal microglia. Further study into the fate of TDP-43 and whether microglial function is altered following TDP-43 uptake in the zebrafish model may help expand upon the early evidence for microglial involvement in the progression of ALS/FTLD [8, 35, 46]. For example, phagocytosis of degradation-resistant TDP-43 aggregates [69] may disrupt the lysosomal pathway in microglia and, therefore, impair their ability for clearance of TDP-43. Interestingly, Paolicelli et al. have recently reported that TDP-43 depletion in Cx3Cr1-expressing cells (including microglia) led to an increase of phagocytosis and elevated transcription of lysosomal components [46]. This suggests that both endogenous and exogenously acquired TDP-43 may alter the functional behaviour of microglia, and contribute towards the progression of ALS/FTLD.

Depletion of microglia through PU.1 knockdown allowed us to visualise intracellular spreading of TDP-43. In fact, we observed at least three pathways for TDP-43 dispersal following motor neuron degeneration after injury: this included diffusion of TDP-43 from the nucleus to the cytoplasm after neuronal swelling, compartmentalised TDP-43 release in discrete quanta, and the breakdown of homeostatic TDP-43 exclusion from the axon. Concerning the interesting axonal spreading of TDP-43 it is important to note that we consistently did not detect any eGFP-TDP43^WT^ inside axons of healthy spinal motor neurons. Neuronal cell cultures and drosophila imaging have identified axonal TDP-43 as a component of transport vesicles in keeping with a role as a chaperone of RNA transport [2, 19]. We frequently observed the cytoplasmic mTagBFP and the CAAX-tagged membrane-bound mKO2 fluorophores incorporated into transport vesicles moving antero- as well as retro-grade along the axon (data not shown). However, we never observed this active transport for eGFP-TDP43^WT^. This difference indicates the intriguing possibility that the incorporation of TDP-43 into transport vesicles may be differentially regulated between neuronal cell types, in an in vivo setting (i.e. cell culture vs vertebrate spinal cord) or in distinct environmental or developmental contexts.

This study provides the first live-imaging data to support axonal-mediated spreading of TDP-43 [9, 45]. In combination with observations obtained in cell cultures suggesting TDP-43 transfer via the axonal membrane [21], this provides evidence that axonal TDP-43 might be an important mediator of ALS/FTLD pathology. It is worth noting that alongside our observations of spinal microglia engulfment of motor neuron cell bodies in this study and in two previous studies [40, 41], we have never observed microglial phagocytosis of degenerating axons. Given that microglial neuronophagia preceded TDP-43 nucleo-cytoplasmic transport and that the anterograde movement of TDP-43 was a late process in neuron degeneration (that we could observe best when microglia were depleted), it is reasonable to conclude that rapid microglial phagocytosis of injured motor neuron somata is an effective mechanism for the prevention of TDP-43 spread.

Taken together, nucleo-cytoplasmic transport of TDP-43 was studied in real time in the living spinal cord, and revealed that depletion of functional microglia leads to axonal and extracellular spreading of the pathological protein. This provides new insight into the potential direct role of microglia as well as pathways for protein distribution in the cascade of cellular and molecular events that cause ALS and FTLD.

## Electronic supplementary material

Below is the link to the electronic supplementary material.

Supplementary material 1 (MP4 27379 kb) **Online Resource 1** Video of motile TDP-43 accumulations in the motor neuron nucleus. 8 kHz resonance scan of a motor neuron cell body with nuclear eGFP-TDP43^WT^ (green) and cytoplasmic mTagBFP (magenta). Scale=1 µm

Supplementary material 2 (MP4 5934 kb) **Online Resource 2** Video of spinal microglia (mCherry-CAAX, red) directed migration toward and engulfment of eGFP-TDP43^WT^ expressing motor neuron during UV-induced degeneration. Scale=10 µm

Supplementary material 3 (MP4 3963 kb) **Online Resource 3** Video of spinal microglia (mCherry-CAAX, red) migration toward an eGFP-TDP43^wt^ expressing motor neuron after UV stress. The neuron does not undergo cell death or demonstrate morphological signs of degeneration over the follow-up imaging period. The microglial cell approaches and makes contacts with the targeted cell and surrounding region, as well as appearing to phagocytose a nearby cell. The microglial cell finally migrates away from the target cell. Scale=10 µm

Supplementary material 4 (MP4 16244 kb) **Online Resource 4** Video of the expansion and dissolution of a motor neuron and its TDP-43 expression over the course of UV-induced degeneration. mTagBFP is expressed in the cytoplasm and eGFP-TDP43^WT^ is initially localised predominantly to the nucleus. Scale=2 µm

Supplementary material 5 (MP4 2567 kb) **Online Resource 5** Video showing the blebbing of a motor neuron cell body over the course of UV-induced degeneration. Nuclear fragmentation and cytoplasmic extrusions occur during the degeneration process. Scale=2 µm

Supplementary material 6 (MP4 13465 kb) **Online Resource 6** High time resolution video of nuclear eGFP-TDP43^WT^ redistribution to the cytoplasm during cell dysmorphia. An 8 kHz resonance scan shows the rapid redistribution of TDP-43 into the cytoplasm. Segments of the axon become visible as eGFP-TDP43^WT^ localises to these regions. Scale=2 µm

Supplementary material 7 (MP4 2416 kb) **Online Resource 7** Video of TDP-43 fragments released from a neuron undergoing UV-induced degeneration. Scale=2 µm

Supplementary material 8 (MP4 1597 kb) **Online Resource 8** Video of progressive fragmentation of the axon in a motor neuron in the spinal cord occurring during UV-induced degeneration. The axon is visualised by a membrane bound mKO2-CAAX (yellow). Scale=10 µm

Supplementary material 9 (MP4 3045 kb) **Online Resource 9** Video of axonal TDP-43 redistribution during UV-induced degeneration. Top panel: Max projection of stacks for eGFP and mTagBFP channels. Fluorescence intensities have been optimised to highlight the axonal distribution of TDP-43. Scale=5 µm. Bottom panel: Fluorescence intensity of eGFP in segments along axon trajectory in 3D over the stack

Supplementary material 10 (PDF 1304 kb) **Online Resource 10** Supplementary figures 1-4
