# Supplementary material for: Nucleo-cytoplasmic transport of TDP-43 studied in real time: impaired microglia function leads to axonal spreading of TDP-43 in degenerating motor neurons
Source: Acta Neuropathol. 2018 Jun 25;136(3):445–59. doi: 10.1007/s00401-018-1875-2 (PMC6096729; doi:10.1007/s00401-018-1875-2)
Supplement: Supplementary file 10 — Supplementary material 10 (PDF 1304 kb) Online Resource 10 Supplementary figures 1-4 [file 401_2018_1875_MOESM10_ESM.pdf]

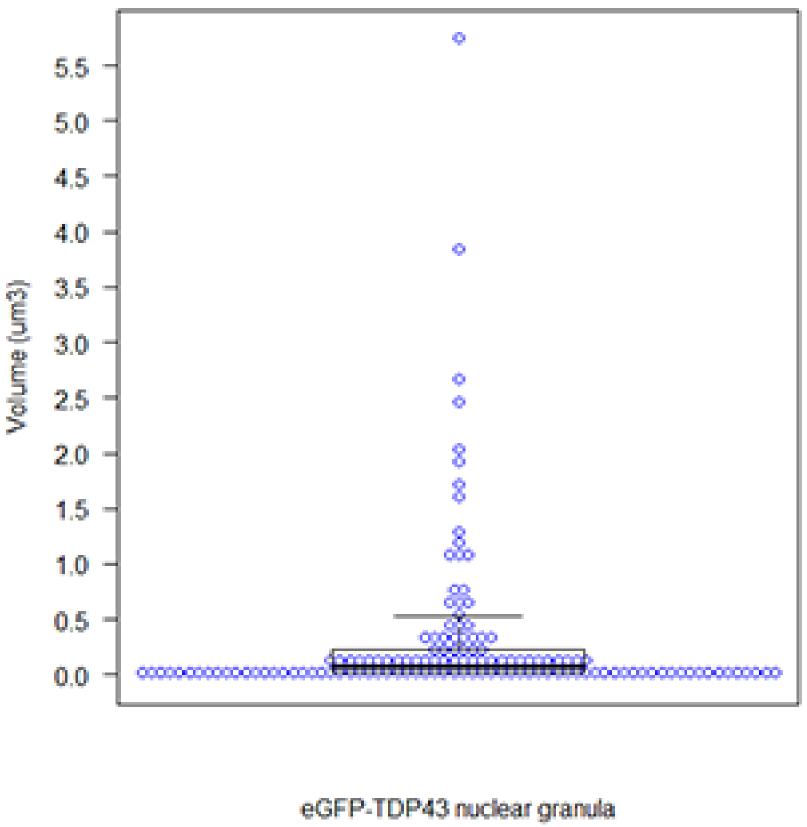

Supplementary Fig. 1 Size distribution of eGFP-TDP43wt granula. eGFP-TDP43wt was not uniformly distributed across the motor neuron nucleus but frequently occurred in motile granula. Each dot represents the volume of the observed granula in the nuclei of zebrafish spinal motor neurons (n=132 granula)

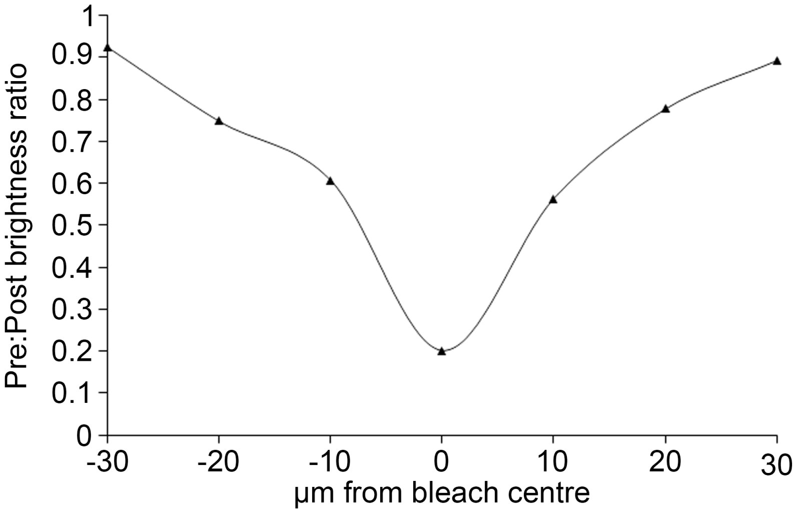

Supplementary Fig. 2 Bleaching curve for region surrounding laser target core. The distance from the ablation site was plotted against the fluorescence ratio, demonstrating the steep laser attenuation >10 µm away from the ablation site.

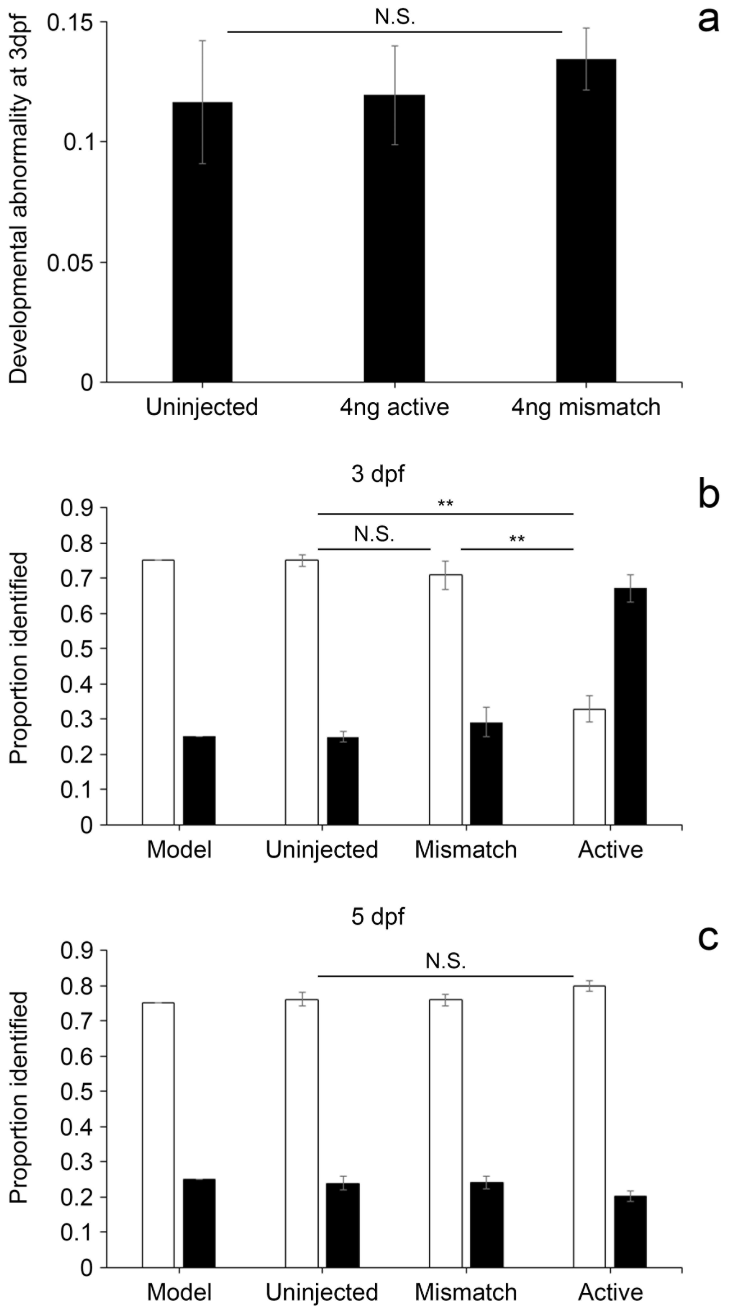

Supplementary Fig. 3 Off-specific effects and efficacy of zebrafish PU.1 morpholino. **a)** Blinded assessment of developmental abnormalities and mortality showed no difference between the active, mismatch injected larvae and uninjected siblings (One-way ANOVA, f(2,9)=0.057, p=0.945, Tukey post-hoc t test, uninjected vs. mismatch p=0.99; uninjected vs. active, p=0.95; mismatch vs. active, p=0.96). **b and c)** Blinded assessment of larvae at 3dpf (b) and 5dpf (c) for presence of mCherry expressing cells in active, mismatch morpholino or uninjected siblings in an *Tg (mpeg1.1:Gal4, UAS;mCherry-CAAX)* incross. White and black bars indicate larvae with or without identified mCherry-CAAX expression, respectively. The morpholino effectively knocked down mpeg1 expressing cells at 3 dpf (One-way ANOVA, f(2,9)=12.51, p=0.003. Tukey post-hoc t test, uninjected vs. mismatch p=0.855; uninjected vs. active, p=0.003; mismatch vs. active, p=0.007). At 5 dpf mpeg1 expressing cells were beginning to recover and there was no difference between groups (One-way ANOVA, f(2,9)=0.229, p=0.8. Tukey post-hoc t test, uninjected vs. mismatch p=0.79; uninjected vs. active, p=0.97; mismatch vs. active, p=0.90). n=4 experiments containing 520 larvae. Error bars represent SEM.

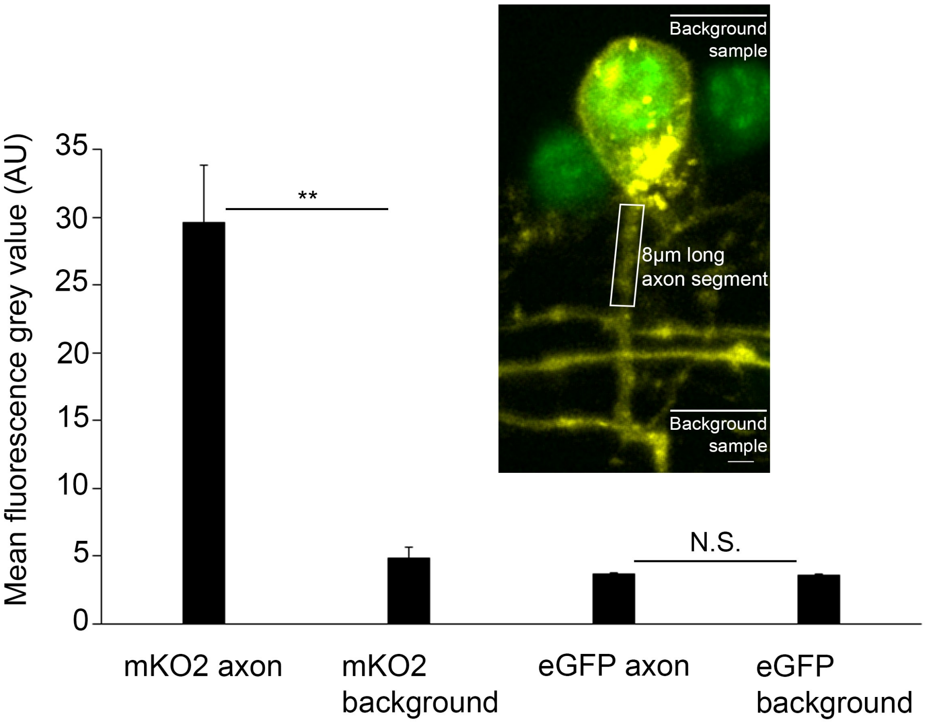

Supplementary Fig. 4 eGFP-TDP43^{WT} was not detected along the proximal axons of healthy motor neurons. Mean fluorescence intensity of mKO2 and eGFP along an 8 µm segment of the axon proximal to the cell body in mKO2-CAAX and eGFP-TDP43^{WT} co-expressers. This was compared to two 8 µm line segments in dark background regions. mKO2 was clearly detected along the axon segment versus control segments ($p=0.002$, Student's t-test) however eGFP did not appear to rise above background $p=0.74$, Student's t-test). Error bars represent SEM. The insert depicts a visual representation of this analysis.
